# Obesity and gut–microbiota–brain axis: A narrative review

**DOI:** 10.1002/jcla.24420

**Published:** 2022-04-14

**Authors:** Arezoo Asadi, Negar Shadab Mehr, Mohamad Hosein Mohamadi, Fazlollah Shokri, Mohsen Heidary, Nourkhoda Sadeghifard, Saeed Khoshnood

**Affiliations:** ^1^ 440827 Department of Microbiology School of Medicine Iran University of Medical Sciences Tehran Iran; ^2^ Microbial Biotechnology Research Center Iran University of Medical Sciences Tehran Iran; ^3^ 56941 Student Research Committee Sabzevar University of Medical Sciences Sabzevar Iran; ^4^ Department of Medical Genetics Faculty of Medical Sciences Tarbiat Modares University Tehran Iran; ^5^ 56941 Department of Laboratory Sciences School of Paramedical Sciences Sabzevar University of Medical Sciences Sabzevar Iran; ^6^ 56941 Cellular and Molecular Research Center Sabzevar University of Medical Sciences Sabzevar Iran; ^7^ Clinical Microbiology Research Center Ilam University of Medical Sciences Ilam Iran

**Keywords:** gut–brain axis, obesity, prebiotic, probiotic, review

## Abstract

**Introduction:**

Obesity is a major health problem that is associated with many physiological and mental disorders, such as diabetes, stroke, and depression. Gut microbiota has been affirmed to interact with various organs, including the brain. Intestinal microbiota and their metabolites might target the brain directly via vagal stimulation or indirectly through immune‐neuroendocrine mechanisms, and they can regulate metabolism, adiposity, homoeostasis and energy balance, and central appetite and food reward signaling, which together have crucial roles in obesity. Studies support the concept of bidirectional signaling within the gut–brain axis (GBA) in the pathophysiology of obesity, mediated by metabolic, endocrine, neural, and immune system mechanisms.

**Materials and methods:**

Scopus, PubMed, Google Scholar, and Web of Science databases were searched to find relevant studies.

**Results:**

The gut–brain axis (GBA), a bidirectional connection between the gut microbiota and brain, influences physiological function and behavior through three different pathways. Neural pathway mainly consists of the enteric nervous system (ENS) and vagus nerve. Endocrine pathway, however, affects the neuroendocrine system of the brain, particularly the hypothalamus–pituitary–adrenal (HPA) axis and immunological pathway. Several alterations in the gut microbiome can lead to obesity, by modulating metabolic pathways and eating behaviors of the host through GBA. Therefore, novel therapies targeting the gut microbiome, i.e., fecal microbiota transplantation and supplementation with probiotics and prebiotics, can be a potential treatment for obesity.

**Conclusion:**

This study corroborates the effect of gut microbiome on physiological function and body weight. The results show that the gut microbiota is becoming a target for new antiobesity therapies.

## Graphical abstract

Gut microbiota is a complex system of organisms, mainly different bacterial species, the gut microbiota interacts with the host, affects host systems and organs (e.g. the brain) and modulates the host physiological functions. The interaction between the gut microbiota and the brain, famously known as gut‐brain axis is a bidirectional connection via neural, immune, and endocrine pathways.

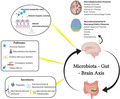

## 1 INTRODUCTION

Currently, obesity is a global concern, and its prevalence has dramatically grown since the past few decades. Based on the World Health Organization's 2016 report, about 1.9 billion of the world's adult population were overweight and 650 million of which were obese. Besides, by 2020, 39 million children aged 0–5 years old will be overweight or obese.^1^ The prevalence of obesity in the world has tripled since 1975, with ~39% of the world's adult population having overweight and 13% having obesity in 2016.^2^

Numerous studies have corroborated the association of obesity with many diseases, such as diabetes, stroke, metabolic disorders, and varied cancer types. It has also been affirmed that obesity can lower the quality of life and can give rise to mental health disorders such as depression and anxiety.^3^ Considering these complications, the management and treatment of obesity are the major issues of concern. Obesity has a complex and multifactorial etiology, and the growing body of largely preclinical studies supports the concept of bidirectional signaling within the gut–brain axis (GBA) in the pathophysiology of obesity, mediated by metabolic, endocrine, neural, and immune system mechanisms.^4^ Gut microbiota is a complex system of organisms, mainly different bacterial species.^5^ The interaction between the gut microbiota and the brain, known as “the gut–brain axis (GBA)” is a bidirectional connection via neural, immune, and endocrine pathways. Signaling from the brain through the autonomic nervous system and the hypothalamic–pituitary–adrenal (HPA) axis influences many gastrointestinal processes, including transit and motility, mucus and fluid secretion, immune activation, intestinal permeability, and relative gut microbial abundance, and gene expression patterns in certain gut microorganisms.^6^ Alterations in the gut luminal environment can affect gut microbial community composition and function.^7^ Through these pathways, the gut microbiota interacts with the host, affects host systems and organs (e.g., the brain), and modulates the host physiological functions, including glucose metabolism and liver function.^8^ Alteration in the gut microbiome composition can potentially associated with various chronic diseases, such as allergic asthma, inflammatory bowel disease, depression, and obesity.^9^, ^10^, ^11^, ^12^ The association of obesity with GBA has hitherto been investigated in numerous studies. A number of research works have attributed the gut microbiota to obesity, owing to the direct modulating action of GBA on the appetite‐related hormones, i.e., leptin (LEP), ghrelin, and glucagon‐like peptide 1 (GLP‐1). It has also been demonstrated that the neural connection of the GBA via the vagus nerve has a key role in changing eating behaviors and appetite.^13^, ^14^ Nonetheless, there is still much to investigate about the physiological mechanism of obesity and GBA and also its effect on appetite, eating behaviors, and other physiological mechanisms of the body. On the other hand, most of these surveys have mostly been performed on animal models. As the gut–microbiota–brain axis has an essential role in modulating obesity‐related behaviors and body function, targeting this pathway is a novel treatment method for the management of obesity.^15^ GBA‐based treatments include probiotic, prebiotic, and synbiotic supplementations and fecal microbiota transplantation.

We review the gut microbiota as a key regulator of host metabolism, central appetite, and its role in metabolic disorders such as obesity, summarize the literature on potential mechanisms of gut–brain axis signaling, review how some bacterial strains might contribute to, or protect against, metabolic disease, and address how FMT and dietary interventions might be novel metabolic therapies in clinical practice in obesity management and discuss some of the recent clinical trials designed in this field.

## 2 OBESITY

Obesity is a medical condition in which the body weight is high above the desirable or acceptable weight, often due to excess accumulated fat in the body. According to body mass index (BMI), a BMI over 30 kg/m^2^ is considered obese, and a BMI greater than 40 kg/m^2^ is defined as morbidly obese.^16^, ^17^ Obesity, as a leading cause of death worldwide, affects a growing number of children and adults. In 2015, 100 million children and 600 million adults in over 195 countries were obese.^18^ As reported before, obesity is correlated with various conditions and diseases, comprising type 2 diabetes mellitus, cardiovascular diseases, obstructive sleep apnea, and osteoarthritis,^3^ suggesting the critical role of obesity in numerous disorders.^19^ Obesity has also been considered a major feature of many syndromes, including Prader–Willi, Cohen, and Bardet–Biedl syndromes.^20^, ^21^, ^22^ There are also several neuroendocrine factors but not related to growth hormone deficiency, pseudohypoparathyroidism, Cushing disease, hypothyroidism, and hypothalamic causes. Among genetic factors, single nucleotide polymorphism (SNP) can impact on obesity‐related genes controlling eating behavior and metabolism. A recent study on obesity has demonstrated the role of SNPs in fat mass, obesity‐associated LEP, and LEP receptor genes.^23^ Besides genetics, a change in epigenetics, such as alteration in microRNA expression, noncoding microRNA, and DNA methylation, is correlated with obesity.^24^, ^25^ Epigenetics, unlike genetics, can be changed throughout the lifespan by lifestyle modification through physical activity, energy intake, and diet.

Obesity occurs in two main distinct processes: resetting the body weight set point and balancing prolonged positive energy.^26^ The first process explains the reason why the studies of effective obesity treatments have faced multiple difficulties.^26^ There are several pathophysiological mechanisms affecting appetite, thus potentially contributing to the development and establishment of obesity. More specifically, appetite is influenced by the interplay between the central nervous system (CNS) and the endocrine system through which signals from peripheral organs, particularly the digestion system, transport to CNS. In other words, LEP and ghrelin are produced peripherally but regulate eating behavior through their action on CNS, especially hypothalamus.^27^ While much work have been conducted to give insight into the mechanisms of fat gain, there are still complex processes required to be clarified.

## 3 GUT–MICROBIOTA–BRAIN AXIS

The gut microbiota is a complex community containing trillions of microorganisms affecting normal physiology and host's susceptibility to disease.^5^ The gut microbiota hosts more than 100 bacterial species, 150 times as many genes as human genome.^5^, ^28^ The gut flora is mainly dominated by bacteria, and by protozoa, viruses, archaea, and fungi.^29^ Gut microbiota composition is a dynamic entity changing throughout the human life depending on the environmental (e.g., diet) and host (e.g., genetics and age) factors.^30^ Gut microbiome possesses many functions; for instance, it maintains intestinal integrity, produces mucus, stimulates intestinal epithelium regeneration, and mediates the production of short‐chain fatty acid (SCFA).^31^ Gut microbiota also mediates the maturation of innate immunity in the early stage of life and somehow performs sensing and modulating enormous amounts of signals from the environment, then behaves throughout the body.^32^ The gut microbiota acts as an intermediate between the host and environment and may potentially influence human health.^33^ An alteration in beneficial bacteria may exert notable effects on the individual's health; in particular, it induces some aspects of disease pathogenesis. Factors such as diet, illness, drug, and infection may also change microbiota.^34^, ^35^, ^36^

**FIGURE 1:**
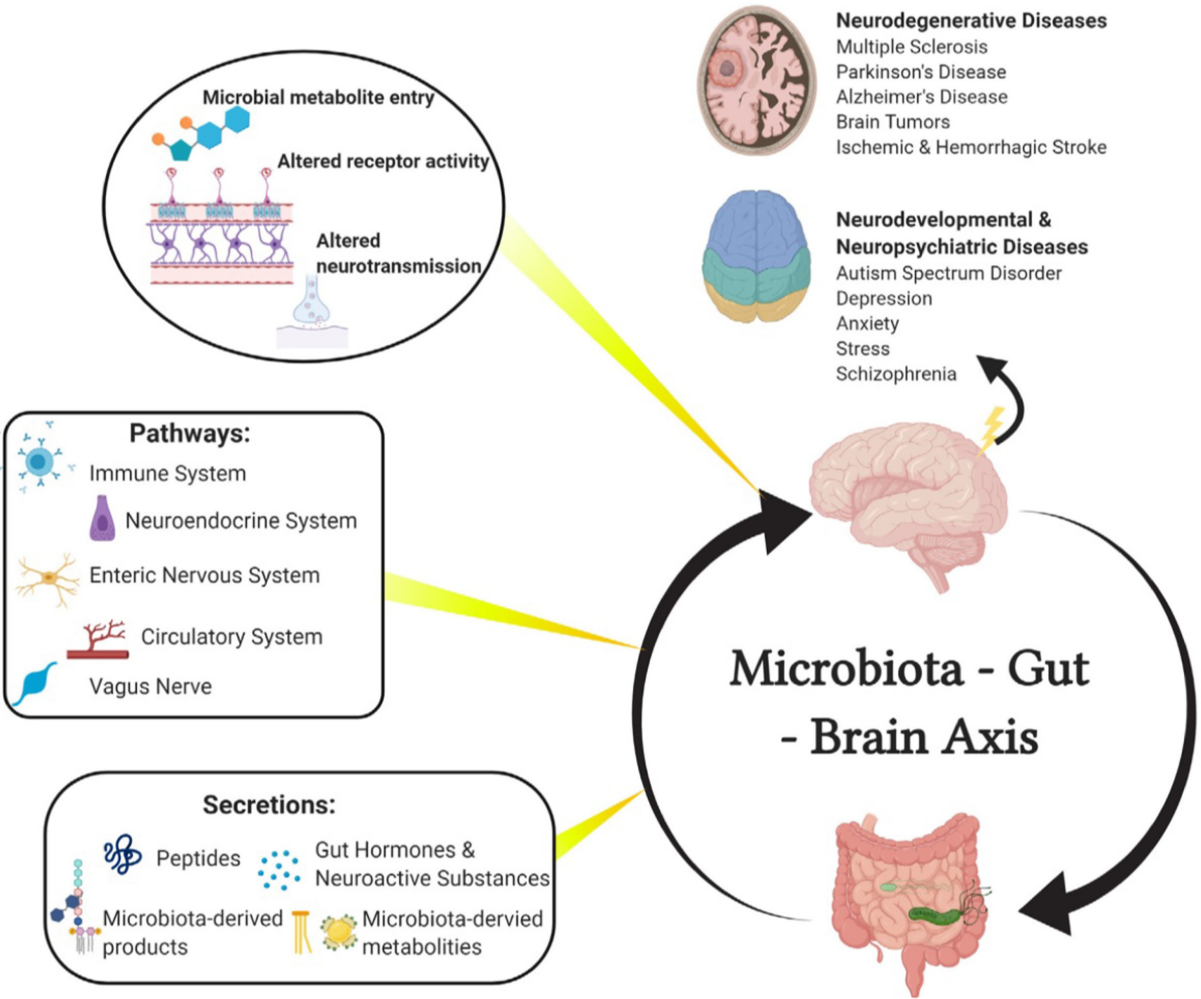
Structure of the gut–microbiota–brain axis (The figure was adopted and reproduced from Liu et al. with permission from the publisher)^116^

The term “gut–microbiota–brain axis” is defined as a bidirectional communication between the brain and gut bacterial community through multiple systems forming a network (Figure 1). It has a significant role in maintaining the homeostasis of the CNS and gastrointestinal system.^37^, ^38^ The interaction pathways in this network include both indirect and direct signaling via the neuronal pathway, immune system, and chemical transmitter, as discussed below.^33^ As different biological systems are engaged in these networks, their complexity needs to be explored.

### 3.1 Neural link between the gut and brain

The nerve fibers physically link the brain to the gut. The vagus nerve and autonomic nervous system (ANS) represent the major neuronal pathways (Figure 1). The former facilitates the bidirectional signals between the gut and brain stem, while the latter innervates the gut and enteric nervous system (ENS). The neuroanatomical routes controlling gut functions form a four‐level integrative organization. The first level is ENS comprising gut glial cells, submucosal ganglion, and myenteric ganglia.^39^, ^40^ The second level, prevertebral ganglia, is the key player in the peripheral visceral reflex response.^39^ ANS, the third level in the spinal cord, brain stem dorsal motor nucleus of the vagus nerve, and nucleus tractus solitarius (NTS), projects and receives the signals from the efferent and afferent fiber of the vagus nerve, respectively.^41^ The fourth level is the higher brain centers. Neuron signals from the cerebral cortex and subcortical area, including funnels and basal ganglia, pass downward to the medulla and pontine nuclei. Once passing through the afferent fiber of the vagus nerve, the NTS transfers to higher centers such as insular cortex, lobus limbicus, and thalamus.^42^

In the afferent pathway, receptors are first involved in the gut tissue. Chemoreceptors are essential for identifying chemical stimuli (hormones and neurotransmitters), and mechanoreceptors are necessary for detecting alterations occurred in the intestinal volume. The vagus nerve, in turn, relays the signal from the gut receptors to CNS.^43^ These gut receptors themselves are influenced by the gut microbiota.^44^ Metabolites produced by the gut bacteria such as SCFA are sensed by a chemoreceptor, i.e., enteroendocrine cells (EECs), leading to calcium signaling that may be transmitted to specific fibers of the vagus nerve in gut epithelium.^45^ The vagus nerve is responsible for downward signals from the CNS to visceral organs and tissues. It also has a role in the normal metabolism and immune system function. These downward pathways to the gut could influence the environment of the gut and eventually exert significant effects on the gut microbiota.^46^ As described above, the vagus nerve provides a prominent channel for signaling both from and to the gut.

In many animal studies, the vagus nerve has been introduced as an important nerve that regulates the behavior of the gut‐brain communication. *Lactobacillus rhamnosus* JB‐1 has been shown to influence the expression of γ‐aminobutyric acid (GABA) receptors in the regions of the brain related to emotions and behavior (i.e., the hippocampus and amygdala nuclei) and also modulates the anxiety behavior.^47^ Interestingly, in mice, vagotomy eliminates the influence of *Lacticaseibacillus rhamnosus* JB‐1 on the expression of GABA receptors.^47^ The vagus nerve has been suggested as a mediator of gut effects on mood and behavior and is essential for the efficient impact of *L*. *reuteri* on social behaviors in autism spectrum disorder (ASD) models.^48^ These findings point to the manipulation or activation of the vagus nerve as a possible approach for treating human disease. To support this assumption, the implantation of an electrical device stimulating the vagus nerve has been approved as an efficient treatment for resistant depression and epilepsy.^49^

### 3.2 Immune system as a link between the gut and brain

Among the body organs, gut contains the highest number of immune cells; therefore, the immune system is a critical network between the brain and gastrointestinal tract (the gut) (Figure 1).^50^ Investigations at both structural and cellular levels have signified that germ‐free (GF) animal models predispose to the development of immune deficiency. In GF models, secretory IgA T helper 17, CD4^+^, and CD8^+^ immune cells diminish. Also, a decrease is found in isolated lymphoid follicles, lamina propria, and Peyer's patches. On the other hand, re‐colonization of *Bacteroides fragilis* can maintain immune maturation of gut‐associated lymphoid tissue.^51^ The immune system has also a major function in obesity. Previous surveys have unveiled that both high fat intake and the increased permeability of the gut barrier can lead to lipopolysaccharide (LPS) absorption, ultimately causing endotoxemia.^52^, ^53^ Notably, this endotoxemia could be hindered by some gut bacteria, which restore the gut barrier integrity.^54^ Obesity is linked to neuroinflammatory disorders such as Alzheimer's disease and depression. Obese individuals are more predisposed to develop these types of pathologies; therefore, neuroinflammation may establish these associations.^55^

### 3.3 Endocrine system as a link between the gut and the brain

The gut microbiota can affect many systems and organs of the body, such as lungs, liver, skin, adipose tissue, and brain through the endocrine system. The relationship between the gut microbiota and brain via the endocrine system is performed through modulating the neuroendocrine system of the brain and also through the production of hormone‐like metabolites by the gut microbiome, which circulates in the body and reaches other organs.^56^, ^57^

The gut microbiota is able to modify many behaviors, including sexual and social, stress‐related, learning, memory, eating, and obesity behaviors, by influencing the neuroendocrine system of the brain, particularly the hypothalamus–pituitary–adrenal (HPA) axis.^56^ HPA axis, which chiefly reacts to stress‐related conditions, is a vital neuroendocrine pathway responsible for the physiological and developmental functions of the human body.^58^

The connection between the gut–microbiota–brain axis and the neuroendocrine system has not fully been understood. However, it has been attributed to the modulation of the hormonal secretion or to the direct production of bacterial metabolites, namely SCFAs, neurotransmitters, and tryptophan (Figure 1). SCFAs, especially butyric acid and propionic acid, can enter the blood circulation and directly cause some alterations in the brain. These molecules can affect different metabolic pathways, i.e., glucose metabolism, catecholamine synthesis, and immunological pathways such as microglia maturation, thus giving rise to physiological and behavioral changes.^59^, ^60^, ^61^ SCFAs also affect the hormonal secretion of EECs by the activation of some specific G protein‐coupled receptors, direct induction of the glucagon‐like peptide 1 (GLP‐1), release of peptide YY (PYY), and indirect release of ghrelin, which all are responsible for eating behaviors, including satiety, hunger, and appetite mechanisms.^14^, ^62^, ^63^

The gut microbiota produces many neurotransmitters, such as catecholamines, GABA, and tryptophan, which can impact on the hypothalamus, thereby changing the neuroendocrine function.^56^ GABA, which is mostly produced by *Levilactobacillus brevis* and *Bifidobacterium dentium*, can cross the blood‐brain barrier and induces its effects directly.^64^ Many of these neurotransmitters are potentially associated with psychiatric and neurologic diseases, e.g., anxiety, depression, autism, and neurodegenerative disorders.^65^, ^66^

The immunological and endocrine pathways of the GBA are connected to each other in many ways, including the LPS of the Gram‐negative bacteria. These antigens cause immune and neuroendocrine activation following some external factors such as stress and diet.^56^, ^67^ It has been denoted that exposure to LPS in the early weeks of life can induce an increase in ACTH and corticosterone secretion in adulthood in response to stress‐related conditions.^68^

Studies have exhibited a bidirectional relationship between stress‐related response and gut microbiota. It has also been displayed that alteration in the gut microbiome can cause changes in the body's response to stress, and experiencing stressful moments may modify the gut microbiome. The mechanism underlying this condition is the effect of the gut microbiota on the production of glucocorticoid hormones and some immune mediators.^56^, ^69^, ^70^

## 4 ASSOCIATION OF OBESITY WITH GBA

Gut–brain axis has a significant impact on various aspects of physiology, including glucose homeostasis, feeding regulation, gut motility, and appetite. Using this system, therapeutics for many diseases, including T2DM and obesity, have been explored.

**FIGURE 2:**
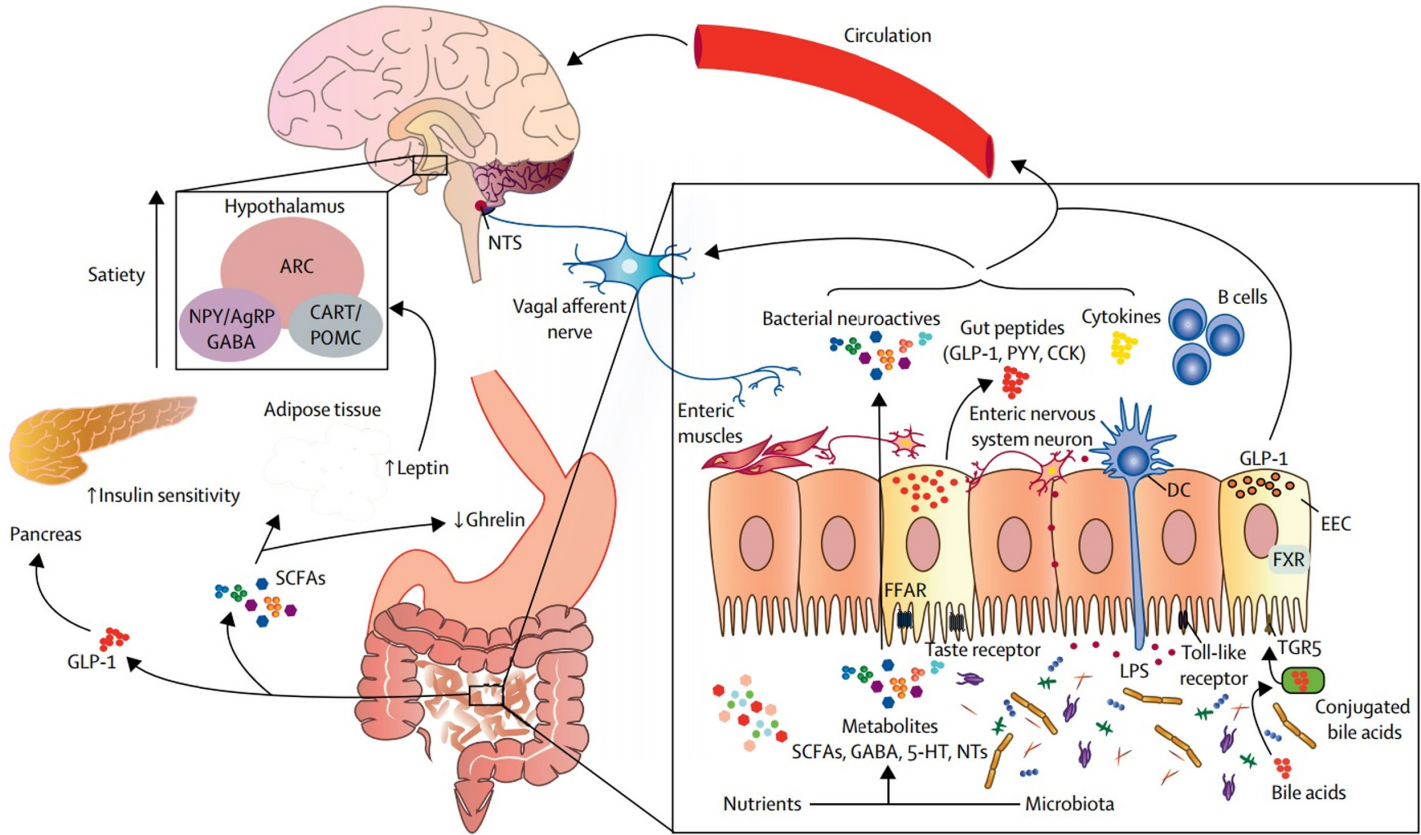
Association of obesity with gut–microbiota–brain axis (The figure was adopted and reproduced from Torres‐Fuentes et al. with permission from the publisher)^53^

An extensive and complex networks of neurons and hormones act bilaterally between the gastrointestinal tract and the brain, and their receptors regulates appetite, food intake, and obesity.^71^ The presence of nutrients in the gastrointestinal tract causes complex hormonal and neural signaling to the brain; the vagus nerve mediates this signaling. Information transmits from the gut to the NTS, and to the smooth muscles of the gut by effector fibers (Figure 2).^14^ Thereafter, information from the NTS distributes to the hypothalamus, a region of the brain that regulates appetite, food intake, and energy balance in the neurons of the arcuate nucleus (ARC). The ARC consists of agouti‐related protein, orexigenic neuropeptide Y, cocaine‐ and amphetamine‐regulated transcript, anorexigenic peptides (LEP), and pro‐opiomelanocortin neurons.^14^ Studies have shown that vagotomy in animal models increases food intake and weight gain by reducing anorexigenic hormone signaling.^72^

Some gut bacteria can modify the secretion of gut hormone, including GLP‐1, ghrelin, PYY, and LEP, thus hypothalamic neuroendocrine pathways affect appetite and satiety.^73^ SCFAs derived from microbiota can bind to receptors on EECs and alter the release of enteric hormones into the systemic circulation.^74^ Moreover, the activation of various taste (bitter, fat, umami, and sweet) receptors in EECs causes the secretion of ghrelin, GLP‐1, and cholecystokinin.^75^ Acetate, the main SCFA secreted by intestinal bacteria, suppresses appetite via central hypothalamic mechanisms.^76^

It is noteworthy that altered microbiota leads to an increase in acetate concentration, thus resulting in the upregulation of the parasympathetic nervous system and also the elevation of increased glucose‐stimulated insulin secretion, ghrelin secretion, and obesity.^77^ Neuroactive metabolites (e.g., serotonin and GABA) affecting the central control of appetite are produced by the gut bacteria.^78^ Common melanocortin neurons, which control bodyweight homoeostasis with serotonin, reduce appetite.^79^ GABA, the main inhibitory neurotransmitter in the CNS is required for the normal regulation of energy balance.^78^

Obesity‐associated microbiota increases the efficiency of calorie uptake from ingested foods.^80^ Thus, an obesity‐associated microbiota in comparison to a lean‐associated gut microbiota provides more energy to the host from other indigestible carbohydrates and proteins by rising the production of different primary fermentation enzymes and nutrient transporters.^81^

## 5 GBA‐BASED OBESITY TREATMENT

The results of surveys have revealed that the gut microbiota has a vital role in the regulation of metabolism, energy homoeostasis, and central appetite in the host. Today, the microbiota is utilized to treat metabolic disorders, like obesity.^82^

### 5.1 Probiotics

**FIGURE 3:**
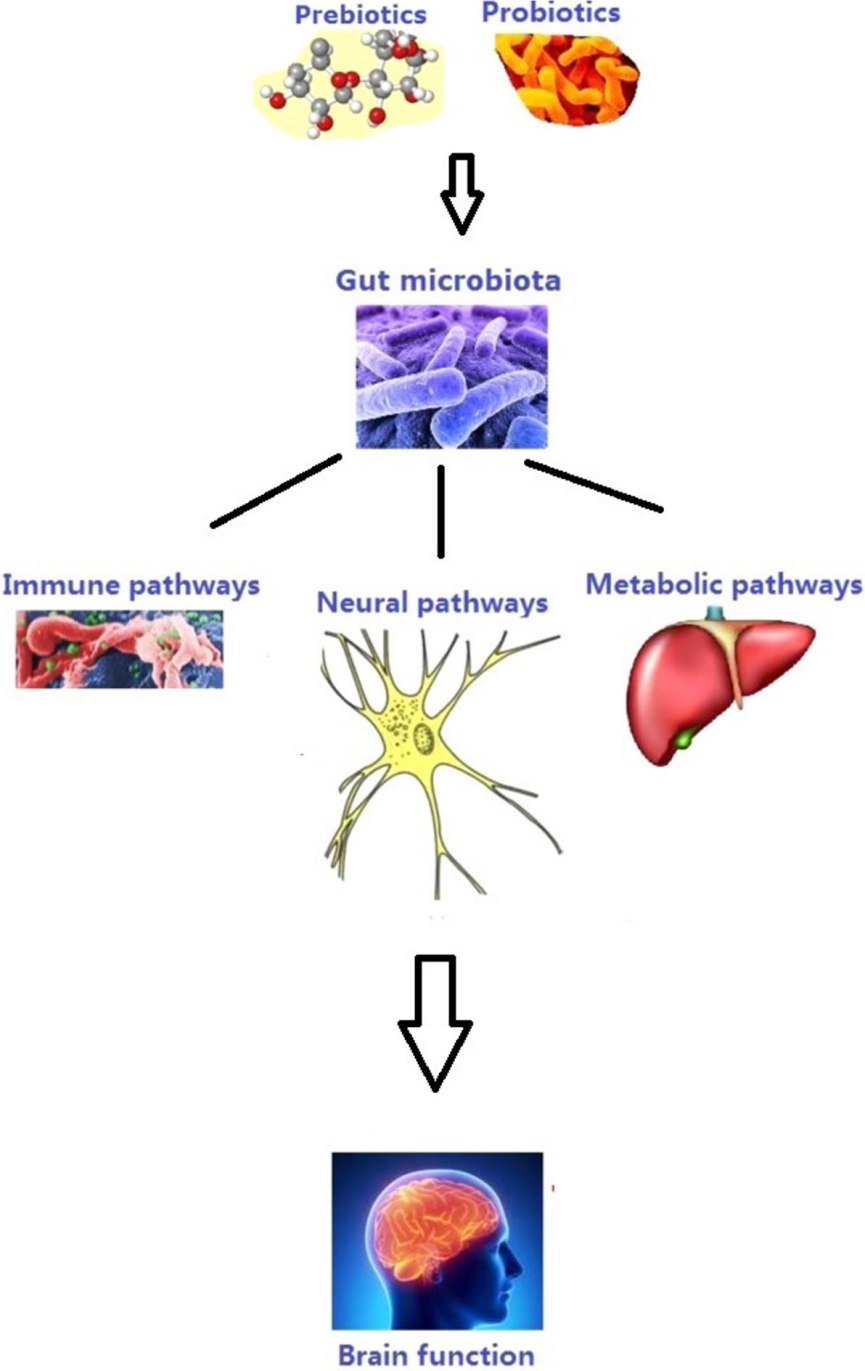
Association of probiotics and prebiotics with gut–microbiota–brain axis (The figure was adopted and reproduced from Liu et al. with permission from the publisher)^117^

Probiotics are “live microorganisms that confer a health benefit on the host when administered in adequate amounts.” Studies have demonstrated a link between probiotics and body weight reduction in animals and humans (Figure 3).^83^ In obese individuals, inappropriate feeding causes a rise in the Firmicutes to Bacteroidetes ratio. This seems to facilitate energy extraction from the ingested food and increases energy storage in the host's adipose tissue.^84^ Probiotics and synbiotics have been proposed to exert a decrease in body weight through different mechanisms. Probiotics help in the recovery of the tight junctions between epithelial cells, thus reducing intestinal permeability, preventing the translocation of bacteria, and reducing inflammation derived from lipopolysaccharides (LPS). The reduction in inflammation leads to an increase in insulin sensitivity in the hypothalamus, which improves satiety. Additionally, increased concentrations of leptin in adipose tissue, glucagon‐like peptide 1 (GLP‐1), and pancreatic polypeptide (PPY) in the intestine lead to a reduction in food intake due to an increase in satiety.^85^

Thus, probiotics can modulate and maintain healthy microbiota. Several bacterial strains have displayed positive effects, such as the reduction of endotoxemia, adiposity, tissue inflammation, bodyweight, LEP levels, and energy intake.^73^ *Bifidobacterium* and *Lactobacillus* spp. are the most common probiotic species that have shown these features; however, their impact on obesity seems to be strain and species‐specific.^86^

### 5.2 Prebiotics

Prebiotics are “selectively fermented ingredients that result in benefits changes in the composition and/or activity of the gastrointestinal microbiota”.^86^ Prebiotics promote the growth of beneficial bacteria in the gut.^87^ These compounds may be considered a future target against obesity. Lactulose, inulin, fructooligosaccharides, and derivatives of galactose and β‐glucans are some typical examples. Oligosaccharides (e.g., insulin), fructooligosaccharides, galactooligosaccharides, and polyphenols are the most widespread prebiotics. These substances may serve as a medium for probiotics, which can stimulate their growth.^88^

The consumption of prebiotics has a significant impact on gastrointestinal microbiota compounds and their metabolic activity. Prebiotics largely affect the modulation of lipid metabolism, the immunological system, enhanced absorbability of calcium, and modification of the bowel function.^89^

Treatment with prebiotics increases the number of helpful bacteria such as *Bifidobacterium* and *Lactobacillus* spp. in the gastrointestinal tract. These bacteria exert effective impacts, comprising of antiobesity, reduced metabolic endotoxemia, decreased circulating proinflammatory cytokines, increased SCFA production, elevated expression of tight junction proteins, and enhanced intestinal barrier function.^73^ The inulin‐type prebiotics promote the growth of beneficial lactobacilli and bifidobacteria, PYY response, and GLP‐1 and also decrease serum ghrelin concentration, which could influence the food intake. The number of beneficial bacteria in patients with obesity and type 2 diabetes is lower than that of healthy individuals.^73^ More research is needed to elucidate the precise mechanisms between prebiotics and obesity, which are still unclear.^90^

### 5.3 Synbiotics

Synbiotics are actually a combination of prebiotics and probiotics and possess synergistic effects. Synbiotics enhance the effectiveness of both prebiotics and probiotics and maximize their beneficial potential for the host's health.^91^ Increasing the survival of probiotics in the gastrointestinal tract is the most important consequence of the production of synbiotics.^92^ Synbiotics exert their positive effects by the provision of specific health impacts and the improved viability of probiotic microorganisms.^93^ Synbiotics modulate the metabolic activity in the intestine through the development of microbiota, the maintenance of the intestinal biostructure, and the inhibition of potential pathogens present in the gastrointestinal tract.^94^ Synbiotics reduce the number of undesirable metabolites such as the inactivation of nitrosamines and cancerogenic substances, in the gastrointestinal tract. Moreover, they elevate SCFAs, carbon disulfides, ketones, and methyl acetates, which potentially result in a positive effect on the host's health.^95^

Synbiotics have several known efficient impacts on humans. They boost the number of beneficial microorganisms, such as *Lactobacillus* and *Bifidobacterium*, and balance the intestinal microbiota.^96^ They also improve hepatic function in patients with cirrhosis. Synbiotics ameliorate the immune system function, inhibit bacterial translocation, and reduce the incidence of nosocomial infections in patients postsurgical procedures.^97^ It appears that synbiotics are highly efficient in the reduction of blood sugar and fat levels, prevention of osteoporosis, and treatment of brain disorders associated with the abnormal hepatic function.^98^

### 5.4 Akkermansia muciniphila

*Akkermansia muciniphila* (*A. muciniphila*) is a Gram‐negative, oval‐shaped, and nonmotile, chemoorganotroph and anaerobic microorganism. It is one of the most famous microorganisms in the gastrointestinal tract and has special effects, such as adipose tissue inflammation, metabolic endotoxemia, fat mass gain, and insulin resistance on the host.^99^ *A. muciniphila* inoculation could cause the clearance of chylomicrons and triglycerides to avoid acute lipid overload in the circulation. These beneficial bacteria interact with different components of the diet, mainly insoluble fiber, release bioactive metabolites and send the signal to the host via the GBA.^100^ *A*. *muciniphila* has the ability to produce enzymes degrading mucin and to utilize its mucin as a carbon and nitrogen source in the mucus layer of epithelium. This microorganism also modulates basal metabolism. Evidence has disclosed that there is a low number of *A. muciniphila* in patients with T2DM and obese people as gut microbial imbalance plays an essential role in upsetting the body's energy balance. Gut microbiota intervention is a potential therapeutic method for treating obesity‐related metabolic diseases, including hyperglycemia and hyperlipidemia.^101^ The metabolic activity of *A. muciniphila* on the host leads to a change in the endotoxin level and SCFA production, and results in the elevation of fatty acid oxidation in the intestine and adipose tissue. *A. muciniphila* converts dietary fiber into butyrate, propionate, and acetate; these metabolites influence glucose and lipid homeostasis. Therefore, *A. muciniphila* can be considered a promising prebiotic from the improvement of metabolic syndromes, such as obesity.^102^

### 5.5 Fecal microbiota transplantation

Direct microbiome therapies, including fecal microbiota transplantation (FMT), is a novel therapeutic option for metabolic syndrome and obesity. FMT is an extremely effective method in which the gut microbiota composition is transferred from a healthy donor to the patient's intestinal tract, typically by duodenal endoscopy or colonoscopy, normalizing the structure and function of the gut microbial community.^103^ After performing FMT, donor microbial strains in human beings are involved in the colonization of the recipient gut microbiota and persist for at least three months.^104^ However, donor‐recipient compatibilities are vital for the successful establishment of the donor's microbial strains in the recipient's gut.^104^ The aforesaid approach has been very successful in treating *Clostridium difficile* infection, with more than 90% efficacy.^105^ Given such a satisfactory effect, it can be expected that FMT can be useful in replacing effective gut microbiota and improving their performance for other diseases in which gut microbiota dysbiosis is involved, such as chronic constipation, irritable bowel syndrome, Crohn's disease, and ulcerative colitis. Moreover, there is increasing evidence that FMT might also have the potential to treat obesity and associated disorders such as type 2 diabetes.^103^

When the composition of gut microbiota is transferred, often by duodenal endoscopy or colonoscopy, from healthy donors with a normal BMI to obese individuals diagnosed with type 2 diabetes, fecal microbiota diversity, and butyrate‐producing bacteria rise.^106^ Of course, FMT can have some potential risks such as the spread of transmissible disease. Although there have been some mild effects such as diarrhea and fever, no side effects have been reported.^107^

## 6 CLINICAL TRIALS

So far, various studies have focused on the effects of probiotics, prebiotics, and synbiotics on human health. There are several clinical trials in this area.

In a study conducted by Larsen et al.,^108^ 50 obese patients were randomized to intake *Ligilactobacillus salivarius* Ls‐33 or placebo for 12 weeks. The fecal microbiota and concentrations of fecal SCFAs were assessed before and after the intervention. Ratios of *Bacteroides*‐*Prevotella*‐*Porphyromonas* group to *Firmicutes* bacteria and *Roseburia intestinalis* significantly increased after the administration of Ls‐33. In a double‐blind, placebo‐controlled trial study 87 subjects with high BMI were selected and randomized to intake *L. gasseri* SBT2055 or placebo daily for 12 weeks. The results represented that BMI, abdominal visceral fat area, and hip circumference decreased.^109^ Another study of 40 obese adults who took *Lactiplantibacillus plantarum* for three weeks, a reduction was observed in BMI and arterial BP values.^110^ Zarrati et al.^111^ performed a survey on 75 healthy overweight and obese individuals randomized to intake *L. acidophilus* La5, *Lacticaseibacillus casei* DN001, *B*. *lactis* Bb12, or placebo for eight weeks. These strains changed the gene expression in the peripheral blood mononuclear cell, and altered BMI, fat percentage, and LEP levels. In Parnell and Reimer's double‐blind, placebo‐controlled trial, 48 healthy adults with a BMI (in kg/m2) >25 were given oligofructose or placebo daily for 12 weeks. Their results showed that body weight was reduced by 1.03 ± 0.43 kg with oligofructose supplementation, while the control group showed a weight gain of 0.45 ± 0.31 kg over 12 weeks (*p* = 0.01). In addition, glucose increased in the control group and decreased in the oligofructose group between the initial and final tests (*p* ≤ 0.05). Oligofructose supplementation did not affect plasma active glucagon‐like peptide 1 secretion. According to a visual analog scale designed to assess side effects, oligofructose was well tolerated.^112^ An earlier study investigated the impact of a combination of *L. rhamnosus* CGMCC1.3724 (LPR) with oligofructose and inulin on 153 obese men and women for 36 weeks. The final outcome was weight loss and decrease in LEP and an increase in Lachnospiraceae.^113^ Another investigation examined the impact of *L*. *casei*, *L. rhamnosus*, *S. thermophilus*, *B. breve*, *L. acidophilus*, *B. longum*, and *L. bulgaricus* combined with FOS on 70 children and adolescents with high BMI for eight weeks. Results suggested a reduction in BMI z‐score and waist circumference.^114^ In an open‐label, randomized, controlled study on 77 obese children, Ipar et al.^115^ implied some promising effects of *L. acidophilus*, *L. rhamnosus*, *B. bifidum*, *B. longum*, *and E. faecium* in combination with FOS. The supplement tested had an effective impact on anthropometric measurements and could decrease TC, LDL‐C, and total oxidative stress serum levels.

## 7 CONCLUSION

This study attempted to review the link between gut–microbiota–brain axis and obesity and discuss GBA‐based antiobesity treatments. According to BMI, a BMI over 30 kg/m^2^ is considered obese, and a BMI greater than 40 kg/m^2^ is defined as morbidly obese. Obesity is correlated with various conditions and diseases, comprising type 2 diabetes mellitus, cardiovascular diseases, obstructive sleep apnea, and osteoarthritis. Gut microbiota is a complex system of organisms, mainly different bacterial species, the gut microbiota interacts with the host, affects host systems and organs, and modulates the host physiological functions. GBA is a bidirectional connection via neural, immune, and endocrine pathways. The evidence base for interventional approaches, which have been shown to affect the composition and function of the intestinal microbiome, includes dietary strategies, oral probiotic/ prebiotic/synbiotic treatment, fecal microbiota transplantation, and bariatric surgery. Targeting the gut microbiota composition can be a potential treatment for obesity. However, further research is necessary to verify the effectiveness and efficiency of this therapeutic approach.

## CONFLICT OF INTEREST

The authors declare that they have no competing interests.

## AUTHOR CONTRIBUTION

Arezoo Asadi, Negar Shadab Mehr, Mohamad Hosein Mohamadi, Fazlollah Shokri, Mohsen Heidary, Nourkhoda Sadeghifard, and Saeed Khoshnood contributed to revising and final approval of the version to be published. All authors agreed and confirmed the manuscript for publication.

## Data Availability

All the data in this study are included in the manuscript.
